# Sex Pheromones of *C*. *elegans* Males Prime the Female Reproductive System and Ameliorate the Effects of Heat Stress

**DOI:** 10.1371/journal.pgen.1005729

**Published:** 2015-12-08

**Authors:** Erin Z. Aprison, Ilya Ruvinsky

**Affiliations:** 1 Department of Ecology and Evolution, The University of Chicago, Chicago, Illinois, United States of America; 2 Department of Organismal Biology and Anatomy, The University of Chicago, Chicago, Illinois, United States of America; Harvard University, UNITED STATES

## Abstract

Pheromones are secreted molecules that mediate animal communications. These olfactory signals can have substantial effects on physiology and likely play important roles in organismal survival in natural habitats. Here we show that a blend of two ascaroside pheromones produced by *C*. *elegans* males primes the female reproductive system in part by improving sperm guidance toward oocytes. Worms have different physiological responses to different ratios of the same two molecules, revealing an efficient mechanism for increasing coding potential of a limited repertoire of molecular signals. The endogenous function of the male sex pheromones has an important side benefit. It substantially ameliorates the detrimental effects of prolonged heat stress on hermaphrodite reproduction because it increases the effectiveness with which surviving gametes are used following stress. Hermaphroditic species are expected to lose female-specific traits in the course of evolution. Our results suggest that some of these traits could have serendipitous utility due to their ability to counter the effects of stress. We propose that this is a general mechanism by which some mating-related functions could be retained in hermaphroditic species, despite their expected decay.

## Author Summary

The *Caenorhabditis elegans* metabolome contains over a hundred ascaroside molecules. Most of them have no known function, or no function at all, but some act as pheromones. Two of these molecules, ascr#10 and ascr#3, are produced in different proportions by males and hermaphrodites. We report that when a hermaphrodite senses a male-specific mixture of these molecules, it changes several aspects of its reproductive physiology, including signaling that guides sperm toward oocytes. During evolution from an ancestor that had both males and females, *C*. *elegans* hermaphrodites lost several female-specific traits, but their reproductive system retained the ability to respond to male pheromones. This greatly aids them during recovery from heat stress. We suggest that serendipitous side benefits of female-specific traits could be a general cause of their retention during evolution.

## Introduction

Comprehensive understanding of any organism requires bridging the knowledge of laboratory biology and natural history [1]. These two approaches to the study of life are complementary and mutually reinforcing, since one offers a powerful methodology for detailed mechanistic investigation, while the other illuminates problems relevant in native evolutionary and ecological contexts. The two aspects of natural history that are often underemphasized in laboratory studies are variation in the environment and interactions among organisms.

A paradigmatic example of a mechanistic study informed by the considerations of natural history is the analysis of dauer formation in *C*. *elegans* [2]. In response to high population density, paucity of food, and other noxious stimuli, L1-stage larvae can enter a morphologically distinct quiescent state that is alternative to reproductive development and is protective against harsh environments. Dozens of genes that control dauer decision have been identified; they connect several signaling pathways and other conserved molecular processes with a flexible reaction to changing environmental conditions. Pheromones, secreted substances that potentiate behavioral or physiological responses, play prominent roles in dauer decision as they communicate information about the number and possibly the status of other worms in the population [3, 4].

We believe that detailed analyses of the physiological and molecular mechanisms that allow worms to cope with other stresses could similarly uncover important biological phenomena. In their native habitats, *C*. *elegans* likely experience frequent and substantial temperature fluctuations [5, 6]. We have studied the mechanisms used by worms to thrive under these conditions [7, 8]. Here we specifically focus on the role of pheromone-mediated communication among animals exposed to prolonged heat stress.

## Results

### Sex-specific ascaroside pheromones improve reproductive recovery from heat stress

**Fig 1:**
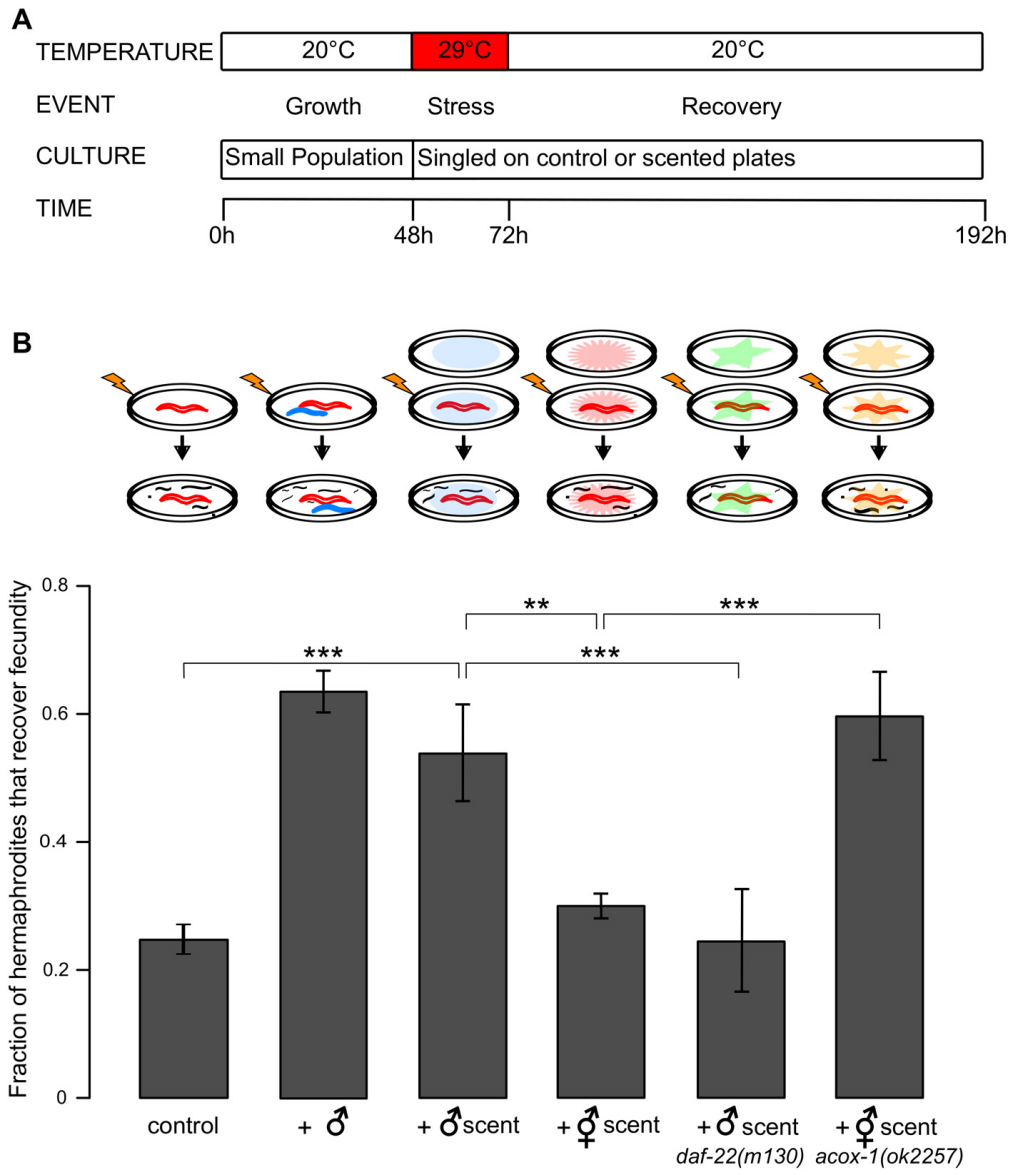
Male scent, likely mediated by ascaroside pheromones, facilitates recovery of fecundity after heat stress. (A) The protocol for heat stress and recovery experiments is summarized. At 48 hours post L1 arrest (young adulthood) hermaphrodites were singled onto either control or scented plates. Unless otherwise indicated, worms were exposed to scent during both stress and recovery. (B) The fraction of hermaphrodites that recover fecundity after 24 hours of heat stress at 29°C. A schematic is depicted above each condition. **P < 0.01, ***P < 0.001. P-values were calculated using binomial test and Bonferroni corrected for multiple comparisons. Error bars are ±SD among separate trials. See S1 Table for raw experimental data including numbers of independent trials and worms tested in each trial.

*C*. *elegans* hermaphrodites can recover reproductive ability following even a prolonged exposure to temperatures at which egg laying stops and none of the offspring survive [8]. Importantly, because *C*. *elegans* hermaphrodites are self-fertile, recovery of a single individual could be sufficient to re-establish a population. To explore this process further and to avoid the potentially confounding effects of population density [9–11] we tested the ability of *singled* hermaphrodites to recover (Fig 1A). Only a small fraction of individuals recovered when kept alone (Fig 1B).

In contrast, when one hermaphrodite and one male experienced stress together, the probability of recovery increased more than two-fold. We have previously demonstrated that because the primary cause of fecundity loss post heat stress is sperm death, hermaphrodites recovered much better after mating with unstressed males [8]. In the experiments reported here, matings did indeed occur when males and hermaphrodites were stressed together or when unstressed males were added (S1 Fig). Yet, broods of many of the recovered hermaphrodites lacked males, which are expected if male-supplied sperm yielded extra progeny. This suggested to us that in addition to male sperm, other causes contributed to the better reproductive recovery of hermaphrodites in the presence of males. Importantly, the physical presence of a male was not required to increase the probability of recovery–scent left on a plate was sufficient, that is, hermaphrodites recovered approximately twice as frequently when they experienced stress and recovery on male-scented plates. Hermaphrodite scent may also improve recovery, but only marginally (Fig 1B). These results suggest that a secreted substance produced primarily by adult (S2 Fig) males conveys a signal that improves reproductive recovery from prolonged heat stress. We tested whether exposure to male scent only during stress or recovery had the same effect as continuous incubation on scented plates. Intermediate recovery levels under each of these treatments suggested that their effects were somewhat cumulative (S3 Fig)

Ascaroside pheromones [3, 4, 12, 13] are likely candidates to mediate this scent-improved recovery. No pheromones produced exclusively by individuals of either sex have been reported in *C*. *elegans* so far. However, two ascarosides, ascr#3 (asc-ΔC9) and ascr#10, are produced in different ratios by the two sexes– 4:15 in males and 7:2 in hermaphrodites, respectively [14, 15]. Thus, sex-specific pheromone cocktails, rather than particular molecules, likely constitute the male and hermaphrodite scents in *C*. *elegans*.

Genetic evidence suggests that sex-specific ascaroside signals are involved in reproductive recovery from heat stress. Loss-of-function of *daf-22*, a gene encoding a β-ketoacyl-CoA thiolase [16], abrogates excretion of shorter chain ascarosides, such as ascr#3 and ascr#10 [14, 15, 17]. The scent of *daf-22*(*m130*) mutant males was unable to improve recovery as much as the scent of wild type males (Fig 1B). In contrast, loss of an acyl-CoA oxidase ACOX-1 inverts the ratio of ascr#10 to ascr#3 production in hermaphrodites to make ascr#10 even more prevalent than in males [14, 15, 18]. As expected, the scent of *acox-1*(*ok2257*) hermaphrodites was as potent as that of the wild type males in improving recovery (Fig 1B).

### *C*. *elegans* can distinguish different ratios of ascr#3 and ascr#10

**Fig 2:**
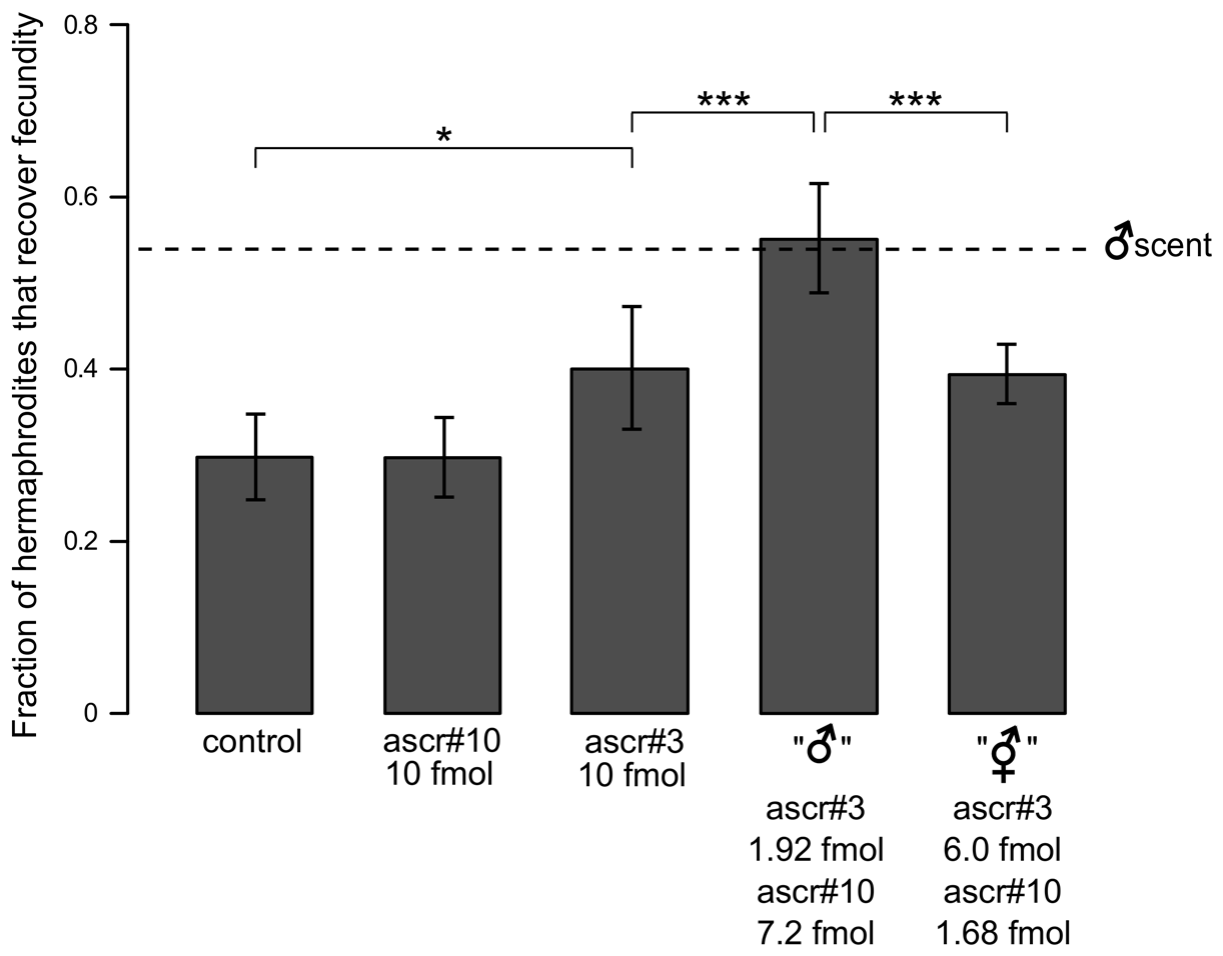
Different ratios of two ascarosides have distinct effects on reproductive recovery from stress. The effect of single ascarosides and cocktails of two ascarosides on the recovery of fecundity after heat stress. *P < 0.05, ***P < 0.001. P-values were calculated using binomial test and Bonferroni corrected for multiple comparisons. Error bars are ±SD among separate trials. See S1 Table for raw experimental data including numbers of independent trials and worms tested in each trial. The dashed line marks the average recovery fraction with male scent (the value shown in Fig 1B). Ethanol on the control plates made recovery somewhat higher than in Fig 1B.

Because male scent improved recovery and ascr#10 is predominantly found in males, we expected that exposure of hermaphrodites to this molecule would increase the probability of fecundity recovery following heat stress. Surprisingly, hermaphrodites on plates scented with purified ascr#10 recovered no better than on control plates (Fig 2).

In contrast, ascr#3, the hermaphrodite-enriched pheromone, improved recovery significantly better, to a level comparable to that induced by the complete hermaphrodite scent. Because both males and hermaphrodites produce ascr#3 and ascr#10, albeit in different quantities, we conducted recovery experiments using cocktails of these molecules. These were concocted to have ascaroside concentrations that matched those produced by a single live animal (male or hermaphrodite) in 24 hours, as reported by Izrayelit et al. [14], because in our experiments single live animals were used to scent plates for 24 hours. Remarkably, simple mixtures of two ascarosides recapitulated the salutary activity of the complete male and hermaphrodite scents, respectively (Figs 1B and 2). The significant difference between the effects of “male” and “hermaphrodite” cocktails suggests that worms can discriminate ratios of ascr#3 and ascr#10. Reinforcing this conclusion, we found that an equal mixture of these two ascarosides, an intermediate between the “male” and “hermaphrodite” cocktails, was no more potent than the “hermaphrodite” cocktail (S4 Fig).

The male scent must be quite strong since a *single* male can produce enough of it in as little as 16 hours (S5 Fig). Although the amounts of ascarosides produced by individual animals were reported previously [14], we examined the effects of a range of concentrations of these molecules on reproductive recovery. ascr#10 had no detectable effect on recovery even at concentrations ~100 times higher than physiological (S6 Fig). In contrast, activity of ascr#3 was dependent on the concentration (S6 Fig). At 2 fmol, the amount produced by a single male in 24 hours [14], it had no discernable effect in our assay, whereas the effect of 10 fmol of ascr#3 (with no ascr#10 added) was indistinguishable from that of the hermaphrodite cocktail. Remarkably, when 2 fmol of ascr#3 was mixed with 7.2 fmol of ascr#10, which alone did not improve recovery (Fig 2), this cocktail was as potent as the complete male scent (Fig 2).

### The male pheromone signal is conserved among Caenorhabditis nematodes

Why do *C*. *elegans* males produce a signal that promotes self-reproduction (evident as improved recovery from heat stress), which presents a direct competition to their own reproductive success? Why does the efficient recovery of hermaphrodite reproduction from stress rely on a signal produced by males, which comprise only ~0.1–0.5% of *C*. *elegans* populations in the wild [6, 19], likely making encounters between the sexes infrequent? We hypothesized that these apparent paradoxes may have an explanation in the evolutionary origin of the *C*. *elegans* mode of reproduction. The few extant hermaphroditic species in the genus Caenorhabditis arose from gonochoristic (male-female) ancestors relatively recently [5]. It is reasonable to assume that in a species in which mating is essential for reproduction, a male-produced signal that alerts the female reproductive system and increases mating efficiency would be advantageous. Recently arisen hermaphroditic species, such as *C*. *elegans*, may have retained elements of this mechanism.

**Fig 3:**
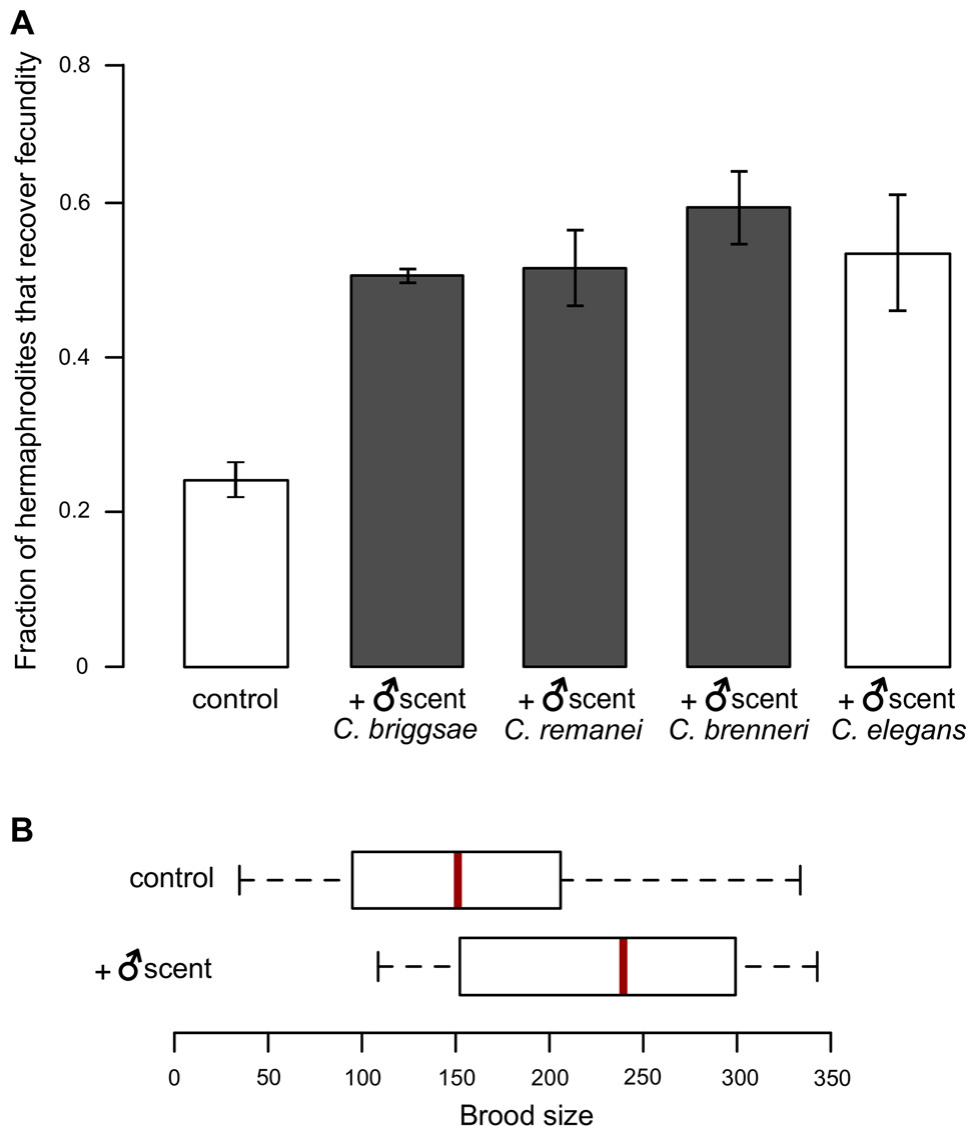
The male pheromone signal is conserved and affects brood size of gonochoristic Caenorhabditis nematodes. (A) Fraction of *C*. *elegans* hermaphrodites that recover self-fecundity after heat stress on plates with male scent of three related species. See S1 Table for raw experimental data including numbers of independent trials and worms tested in each trial. Recovery on control and *C*. *elegans* male-scented plates are given for comparison (shown as white bars); these data are the same as shown in Fig 1B. Error bars are ±SD among separate trials. (B) Brood sizes of *C*. *remanei* females exposed to male scent for 16 hours were significantly higher than naïve females after 10-minute matings (at 20°C) (*P* = 9.5 x 10^−3^, Kolmogorov-Smirnov test). Red lines mark the median values. Means are: control = 160.1 and male-scented = 205. See S2 Table for numbers of independent trials and worms tested in each trial.

We therefore tested whether the male-produced pheromone signal is species-specific. Consistent with the previous finding that ascaroside signaling is broadly conserved in nematodes [20], we found that the scent of males of three species from the genus Caenorhabditis (two gonochoristic and one hermaphroditic) could rescue the reproductive performance of heat-stressed selfing *C*. *elegans* hermaphrodites as well as the scent of the conspecific males (Fig 3). We concluded that despite changes in the reproductive mode, *C*. *elegans* retained the male-specific pheromone signaling system that arose in its gonochoristic ancestor–males still produce the pheromone, while hermaphrodites can respond to it.

### Male scent makes matings more productive in a gonochoristic species

We thought that a likely ancestral function of male pheromones was to facilitate reproduction following mating. For this reason, we compared brood sizes produced by females of a gonochoristic species *C*. *remanei* that were either exposed to male scent or maintained on control plates. Reports of the time for sperm transfer during mating in *C*. *elegans* vary from 4 seconds [21] to 90 seconds [22] with the majority of sperm transferred at the beginning of ejaculation. In addition to measuring the time of sperm transfer during mating (about 17 seconds), Lebouef et al. [23] studied the refractory period in *C*. *elegans* males. They found that males fell into two classes–those with a relatively short refractory period between matings (less than 9 minutes) and those with a longer refractory period between matings (greater than 9 minutes). Males that exhibited a short refractory time were unlikely to produce many progeny from a second mating because they could not produce fresh sperm during the shorter refractory period. We considered these facts and decided that a ten-minute mating period made it most likely that only one successful mating could take place. Incidentally, this period is approximately 1% of the time that is required for male scent to have an effect on the probability of hermaphrodite’s recovery (S5 Fig). At the end of the ten minutes, if the mating pair was still together, the female was gently touched with a platinum wire. If the mating pair did not separate–indicating that mating was ongoing, that female was excluded from the data.

As expected, we found that brood sizes of *C*. *remanei* females that had been exposed to male scent prior to mating were significantly greater than the brood sizes of mated naïve females (Fig 3B). This result strongly argues that the function of the male-specific pheromones in the gonochoristic ancestors of *C*. *elegans* was to alert the female reproductive system and induce appropriate physiological changes that resulted in substantially increased reproductive efficiency upon mating.

### Male scent does not affect brood size in selfing *C*. *elegans*

**Fig 4:**
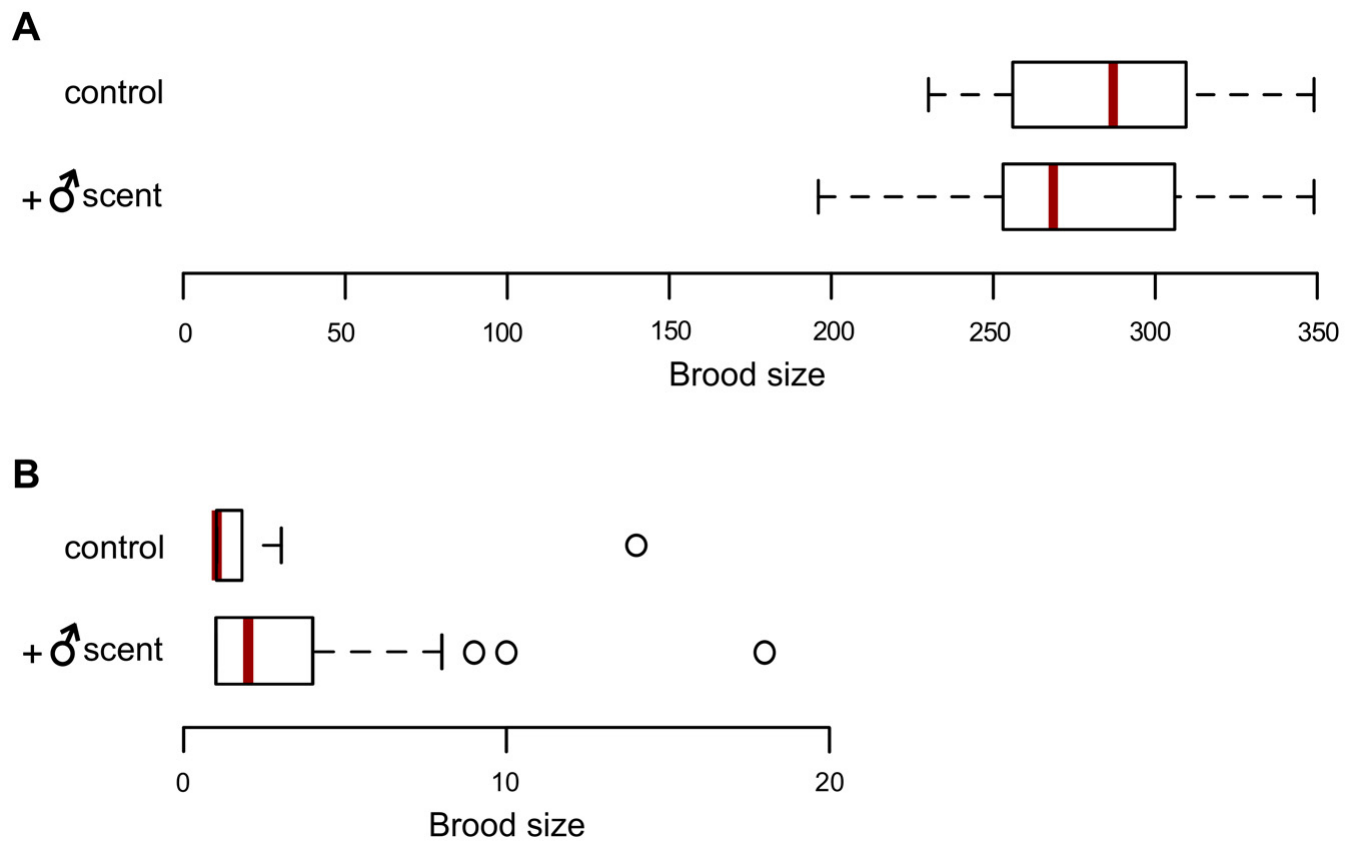
Effects of male scent on brood sizes of selfing hermaphrodites at 20°C and after recovery from heat stress. (A) Self-brood sizes of *C*. *elegans* hermaphrodites raised at 20°C with or without *C*. *elegans* male scent. Broods from 24 hermaphrodites were determined for each condition. Red lines mark the median values. Means are: control = 285.3 and male-scented = 277.3. They are not significantly different (*P* = 0.99, Kolmogorov-Smirnov test). (B) Brood sizes for hermaphrodites recovering from heat stress alone (i.e. recovery is due to self-fertilization) on control or male-scented plates. The animals whose brood sizes were counted were the same as whose recovery is shown in Fig 1B (see S1 Table for raw experimental data). Only worms with offspring were considered. Red lines mark the median values. Means are: control = 2.3 and male-scented = 2.6. They are not significantly different (*P* = 0.27, Kolmogorov-Smirnov test). See S2 Table for numbers of independent trials and worms tested in each trial.

We tested whether the male scent affects brood sizes of *C*. *elegans* hermaphrodites reproducing via self-fertilization at non-stressful conditions (20°C), but found no detectable differences between control and male-scented plates (Fig 4A). Next we wondered whether the two-fold more likely recovery from heat stress in the presence of male scent (Fig 1B) was accompanied by an increase in brood sizes. It was not–brood sizes of *C*. *elegans* recovering via selfing on male-scented plates were not significantly different from those of animals recovering on control plates (Fig 4B and S7 Fig).

### Male scent improves sperm guidance toward oocytes

We considered several possible physiological mechanisms by which pheromone signaling could have improved reproductive recovery following heat stress. Because hermaphrodites were exposed to the 29°C stress after gamete production irreversibly shifted from spermatogenesis to oogenesis, improved recovery in the presence of male scent could in principle be due to a) improved sperm survival/retention of function, b) better preservation of the “female” aspects of the reproductive system including oocytes or gonads, or c) higher post-zygotic survival. Although the catastrophic reduction of brood sizes in hermaphrodites recovering alone from heat stress is primarily due to sperm loss [8, 24, 25], the similar brood sizes of worms recovering on scented and control plates suggested that the increase in the probability of recovery induced by the male scent was due to a more efficient gamete utilization, not their improved survival or post-zygotic mechanisms.

In addition to the catastrophic sperm loss, other elements of the reproductive system also suffered considerable damage during stress. For example, large concretions consisting of oocytes and embryos formed in the uterus [8]. Recovery required purging of these obstacles to allow live fertilized oocytes to pass. This and possibly recovery of the gonad itself required time. Both self recovery and recovery following mating with unstressed males did not yield live L1-stage progeny until ~72 hours post-stress, suggesting that first productive fertilizations occurred ~48–60 hours after the return from 29°C to 20°C (S8 Fig).

We have previously shown that *C*. *elegans* hermaphrodites are able to suppress ovulation when the temperature reaches 31°C and that this improves recovery when the worms are shifted to a more permissive temperature [8]. Therefore, we tested whether male scent suppressed ovulation at 29°C. The numbers of oocytes in the gonad and embryos in the uterus were likewise not appreciably different between these treatments (S9 Fig). We concluded that other mechanisms were likely responsible for the improved recovery.

**Fig 5:**
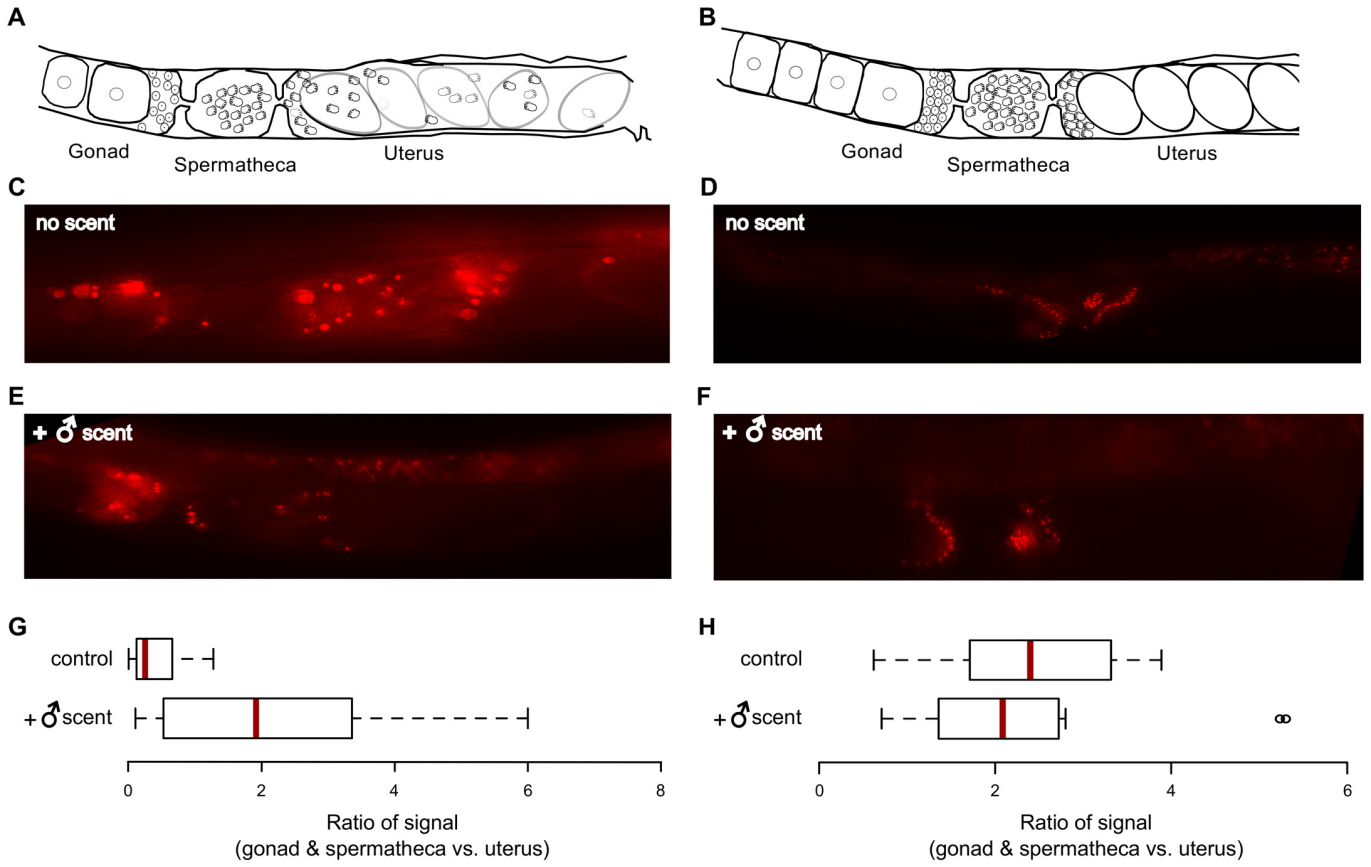
Male scent enhances sperm guidance following stress. Schematic drawing of the gonad of a hermaphrodite stressed at 29°C (A) and unstressed control (B). Representative images of worm gonads (with mCherry-labeled sperm) on control plates after heat stress (C) or at 20°C (D) and on male-scented plates after heat stress (E) or at 20°C (F). For all photographs, anterior is to the left and ventral is down. Quantification of sperm distribution in heat stressed animals grown on control vs. male-scented plates (G) and in unstressed animals (20°C) grown on control vs. male-scented plates (H). Red lines mark the median values. The ratios are significantly different in G (*P* = 1.3 x 10^−4^, Kolmogorov-Smirnov test), but not in H (*P* = 0.79, Kolmogorov-Smirnov test). Red lines mark the median values. Means in G are: control = 0.4 and male-scented = 2.1. Means in H are: control = 2.3 and male-scented = 2.4. See S2 Table for numbers of independent trials and worms tested in each trial.

One phenomenon related to gamete utilization that is modulated by ascarosides has been recently described in *C*. *elegans* [26–28]. Oocytes were shown to secrete a prostaglandin signal that improves sperm targeting to the spermatheca, an organ where fertilization occurs (Fig 5A and 5B). We therefore tested whether sperm guidance was improved on male-scented plates. Using a strain in which sperm were marked with an mCherry reporter gene [29] (recovery of this strain does not appear to be different from that of N2; S10 Fig), we measured the distribution of this fluorescent label in the reproductive tracts of hermaphrodites following heat stress. In worms recovering on control plates most sperm were trapped in the uterus (Fig 5C), likely because continued ovulation under these conditions ([8] and S9 Fig) displaced sperm from the proximal gonad (all spermatids remained in the gonad in the absence of ovulation at 31°C; see S11 Fig). In contrast, we saw evidence of significantly higher localization of sperm in the spermatheca and the proximal gonad in worms recovering on male-scented plates (Fig 5E and 5G). In unstressed animals male scent did not appear to have a detectable effect on sperm guidance (Fig 5D, 5F and 5H). We inferred that under these conditions, guidance of self sperm toward oocytes is sufficiently high and cannot be substantially improved by the pheromone signal.

### ascr#10 and ascr#3 have distinct effects on the reproductive system

**Fig 6:**
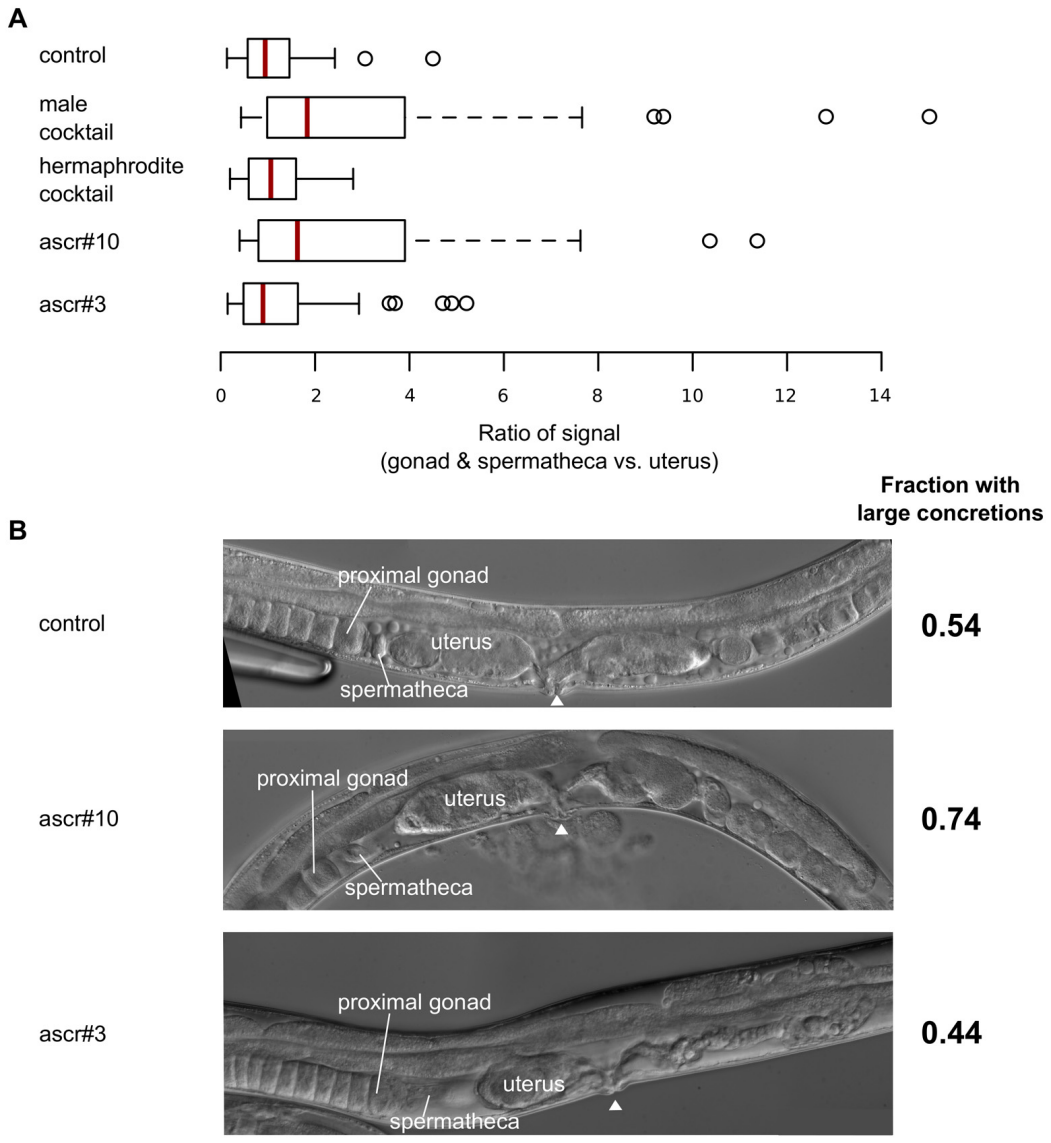
ascr#10 and ascr#3 have different effects on the *C*. *elegans* reproductive system. (A) Sperm guidance in heat-stressed hermaphrodites on plates with individual ascarosides and ascaroside cocktails. Sperm guidance on plates with male cocktail (*P* = 3.1 x 10^−3^, Kolmogorov-Smirnov test Bonferonni corrected for four comparisons) and ascr#10 (*P* = 4.3 x 10^−3^, Kolmogorov-Smirnov test Bonferonni corrected for four comparisons) were significantly different from control. Red lines mark the median values. Means are: control = 1.1, male cocktail = 3.2, hermaphrodite cocktail = 1.2, ascr#10 = 2.8, and ascr#3 = 1.3. (B) Representative photographs of heat-stressed hermaphrodites during recovery. Hermaphrodites were monitored during recovery for the clearance of large concretions formed in the uterus during heat stress. Worms were examined at 48, 72, and 96 hours of recovery–that is, the time corresponding to when most of recovery occurs (See S8 Fig). Worms on plates with ascr#10 were significantly worse at clearing large concretions from the uterus (*P* = 8.7 x 10^−3^, binomial test Bonferroni corrected for two comparisons). In both (A) and (B) individual ascarosides were at 10 fmol. Male and hermaphrodite ascaroside cocktails were as in Fig 2 (1.92 fmol ascr#3 + 7.2 fmol ascr#10 and 6.0 fmol ascr#3 + 1.68 fmol ascr#10, respectively). See S2 Table for numbers of independent trials and number of worms tested in each trial.

To confirm that the improved sperm guidance caused by the male scent was mediated by ascarosides, we tested sperm guidance on plates containing ascr#10, ascr#3, and their mixtures. Sperm guidance was significantly improved on male- but not hermaphrodite-specific cocktail, an effect that appears to be mediated by ascr#10 alone (Fig 6A). Because ascr#3 did not have any discernable effect on sperm guidance and yet somewhat improved recovery (Fig 2), we wondered about its possible mode of action that was distinct from ascr#10. To explore whether it had an effect on the female aspects of the reproductive system, we examined the rates of ovulation and egg-laying, but did not observe any difference between control animals and those on plates containing either ascaroside. We did, however, find that in the presence of ascr#3 many fewer animals had large concretions in the uterus during recovery than did animals on ascr#10 (Fig 6B). Clearing of the reproductive tract is important for the passage of fertilized oocytes, suggesting that ascr#3 contributes to an improved recovery of fecundity via a mechanism distinct from that of ascr#10.

### DAF-7 signaling mediates the effects of male scent on the reproductive system

**Fig 7:**
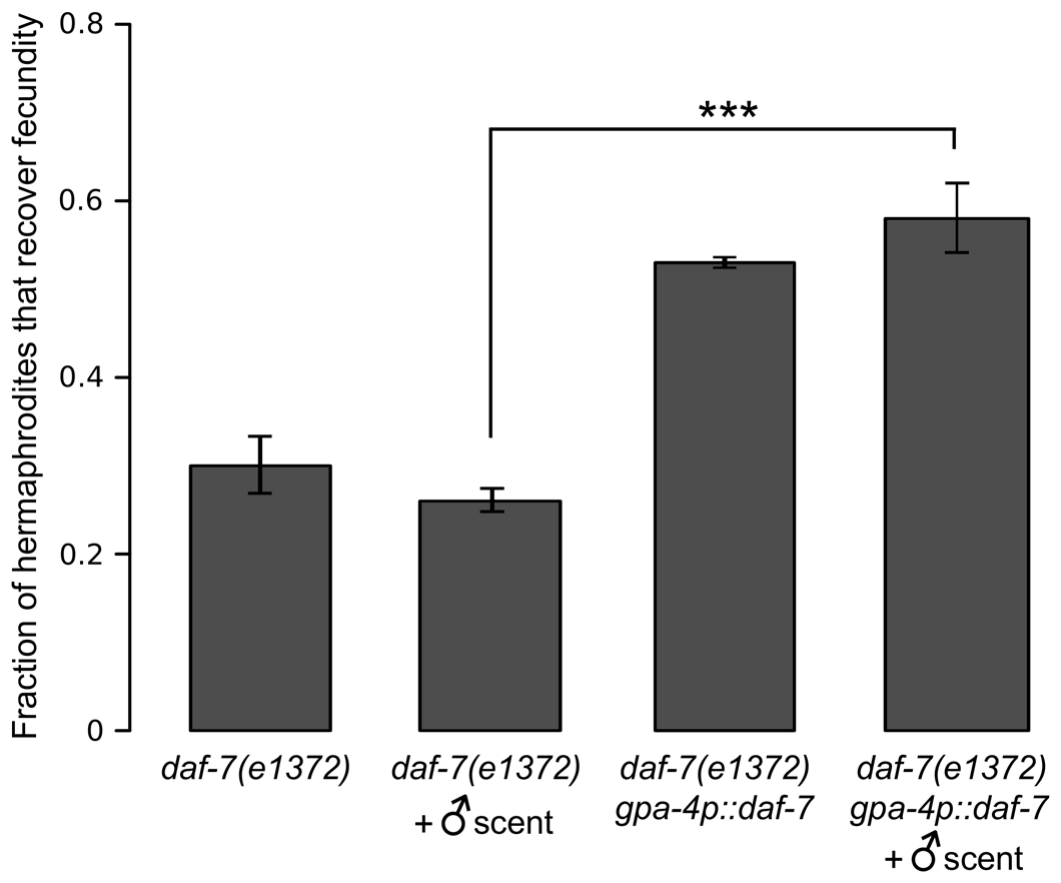
DAF-7 function in ASI neurons is necessary and sufficient for improved reproductive recovery caused by the male scent. The fraction of hermaphrodites that recover fecundity after 24 hours of heat stress at 29°C. *P* = 4.2 x 10^−8^, binomial test. Error bars are ±SD among separate trials. See S1 Table for raw experimental data including numbers of independent trials and worms tested in each trial.

The DAF-7 (a TGF-β-like ligand) function in ASI neurons mediates multiple aspects of *C*. *elegans* response to the environment, including pheromones [30, 31]. In particular, it plays an important role in connecting sperm guidance to environmental conditions [27, 28]. *daf-7* loss-of-function or high concentrations of dauer pheromones ascr#2 and ascr#3 disrupt sperm guidance [28]. We therefore explored the role of DAF-7 signaling on reproductive recovery from heat stress. As expected, we found that *daf-7(e1372)* mutants recovered no better in the presence of male scent than they did on control plates at the frequency comparable to that of N2 on control plates (Fig 7). When the *daf-7* mutation was rescued by constitutively expressing DAF-7 in ASI neurons, recovery was significantly improved, although it still was insensitive to male scent. We interpret these results to mean that DAF-7 signaling is both necessary and sufficient for improved recovery and, in particular, DAF-7 inducibility is required for the response to male scent.

A peculiar feature of the set up of this experiment offered us an additional insight into the complex nature of environmental influences on the reproductive system. The *daf-7(e1372)* allele is temperature-sensitive and worms have to be reared at the permissive temperature of 16°C past the L2-larval stage to avoid the constitutive dauer phenotype [30]. We suspected that this treatment–16°C until late L2 and 20°C until young adulthood–improved the probability of recovery from the 29°C stress (compare *daf-7(e1372)* in Fig 7 and N2 on control plates in Fig 1B). We confirmed that the temperature treatment and not the genotype of the animals was responsible for the observed differences. The N2 worms reared under this regime recovered better than the animals grown constantly at 20°C, whereas *daf-7(e1372)*; *gpa-4p*::*daf-7* grown at 20°C recovered less well than when grown at 16°C until late L2 and at 20°C thereafter (S12 Fig). Cooler temperatures during the first two larval stages evidently make some aspect(s) of the reproductive system more apt to recover from heat stress.

## Discussion

Our results support six conclusions. First, secreted compounds produced primarily by males improve female reproductive performance in a variety of species [32–34]. We showed that this is the case in Caenorhabditis nematodes–male scent potently affected different aspects of female/hermaphrodite reproductive physiology. In a gonochoristic species *C*. *remanei* this resulted in larger brood sizes. In a hermaphroditic *C*. *elegans* it conferred salubrious effects after heat stress. Whereas animals of both sexes produce pheromone cocktails containing multiple distinct molecules [13, 14], we showed that ascr#3 and ascr#10 promote efficient uterine clearing and improve sperm guidance, respectively.

Second, the study of McKnight et al. [28], which demonstrated the effects of environmental cues on sperm guidance, reported that “pheromones” inhibited proper targeting. Here we report an ostensibly opposite result of “pheromones” promoting sperm guidance. We think that two factors could readily explain this apparent contradiction. The pheromones used by McKnight et al. [28] were ascr#2 and ascr#3 (also known as asc-C6-MK and asc–ΔC9, respectively), two main components of the “dauer pheromone” [4, 12]. In contrast, we tested ascr#10 and ascr#3. The amounts of applied pheromones were also different. Whereas we tested concentrations as low as ~2–10 fmol, roughly corresponding to daily production of single animals [14], McKnight et al. [28] reported results using 10 μmol. Taken together, these results suggest a model in which low, male-specific concentrations of ascarosides manipulate the hermaphrodite reproductive system in a way that potentiates improved sperm guidance, whereas high concentrations of hermaphrodite pheromones (reflecting overcrowding) disrupt sperm guidance.

Third, the importance of relative concentrations of ascarosides is highlighted by the curious mode of action of ascr#3 and ascr#10. In previously described synergistic interactions between ascarosides, each molecule had some effect, while the combined effect was greater [12, 35, 36]. The male-enriched ascr#10 alone has no discernable effect on the reproductive recovery from stress, whereas the hermaphrodite-enriched ascr#3 has a modest effect. This is all the more remarkable considering that the two molecules are nearly identical, the only difference between them being one double bond [14]. The synergy between these two molecules depends on their relative concentrations–a “male-like” ratio of ~15:4 (ascr#10:ascr#3) greatly potentiates recovery, whereas a “hermaphrodite-like” ratio of ~2:7 (ascr#10:ascr#3) is indistinguishable from the effects of ascr#3 alone. This implies that worms could discriminate not only the presence of specific pheromone molecules, but their ratios as well. This synergy appears to be mediated by the complementary action of two distinct mechanisms–clearing of the reproductive tract (by ascr#3) and sperm guidance (by ascr#10). The phenotypic effects of other pheromone mixtures may be similarly complex. The relationships between concentration and activity may also be different for different functions mediated by ascr#3 and ascr#10 [4, 10, 20]. Still, for example, ascr#10 was active at concentration ~10 fmol in both mate holding [14] and increasing sperm guidance (Fig 6A). Our findings expand the universe of functions previously ascribed to ascr#3 –in dauer formation [4], in attracting males and repelling hermaphrodites [12] and ascr#10 –in attracting hermaphrodites and holding them in place [14].

Fourth, the results of McKnight et al. [28] and our data (Fig 7) suggest that the DAF-7 TGF-β-like ligand in ASI neurons plays a critical role in conveying the signal to the reproductive system of, respectively, the dauer ascarosides (ascr#2 and ascr#3) and male-specific cocktail of ascr#10 and ascr#3. Previous studies documented DAF-7 functions in mediating response to dauer pheromone [30, 31] and male sexual attraction to hermaphrodite pheromones [37]. Together these studies implicate DAF-7 in ASI neurons in mediating multiple aspects of pheromone-influenced behaviors.

Fifth, *C*. *elegans* researchers are well familiar with the fact that cultivation conditions could have profound effects on animal physiology. Dauer formation in response to high density or paucity of food [2] and more subtle consequences of being raised in isolation [9] are prime examples of this. Our finding that male pheromones, even at femtomole concentrations, can have substantial effects on hermaphrodite reproduction raises a note of practical caution–the presence of males could change multiple aspects of hermaphrodite behavior and should thus be considered in experimental design.

Finally, our results suggest a simple scenario for the evolution of this pheromone signaling system. Its original function in the ancestral gonochoristic species was to communicate the proximity of males to females, which facilitated reproductive success following mating (Fig 3). Transition to a self-fertile hermaphroditic mode of reproduction is expected to have changed selection pressures on sex-specific traits [38]. In particular, hermaphrodites do not need to locate mates for reproduction. Whereas Caenorhabditis hermaphrodites lost several “female” functions [39–41], why did they retain the ability to respond to male-specific pheromones, which do not increase brood sizes produced by selfing, a predominant mode of reproduction in the wild [6, 19]? Temperature fluctuations likely routinely expose *C*. *elegans* to chronic heat stress in its natural habitats [6]. This results in drastically reduced brood sizes, but also increased incidence of males among the recovered offspring [42–44]. Hermaphrodites that retained the ability to respond to male pheromones would therefore gain a substantial advantage because of a greatly increased probability of reproductive recovery. Other female-specific functions in recently evolved hermaphroditic species could also have been preserved by co-option due to their serendipitous ability to counteract the effects of stress.

## Materials and Methods

### Strains

N2 *C*. *elegans* WT,

DR476 *daf-22(m130)* II,

VC1785 *acox-1(ok2257)* I,

EG4883 *oxIs318[pCFJ167(Pspe-11*::*mCherry*::*histone–Cbr-unc-119(+))]* II *unc-119(ed3)* II,

CB1372 *daf-7(e1372)* III,

DA2202 *daf-7(e1372*) III; *adEx2202[gpa-4*::*daf-7 + rol-6p*::*GFP]*,

AF16 *C*. *briggsae* WT,

EM464 *C*. *remanei* WT,

PB2801 *C*. *brenneri* WT.

### Maintenance

All strains were maintained at 20°C under standard conditions [45], except *daf-7(e1372)* and DA2202, which were maintained at both 16°C and 20°C for different experiments. Synchronized cultures of L1 larvae were prepared by hypochlorite treatment of gravid hermaphrodites [46]. The liberated eggs were allowed to hatch in M9 Buffer overnight and the arrested L1 larvae were plated the next morning. The time that L1 worms were deposited on plates was noted as “0 hours post L1 arrest”. Between 30 and 50 L1 larvae were transferred to each lawn plate of *E*. *coli* OP50 and hermaphrodites were kept on these small population plates until just before young adulthood (generally, 48 hours post L1 arrest at 20°C).

### Heat stress and recovery experiments

In a typical experiment, to assess one condition, 25 or 50 hermaphrodites were singled onto plates just after the L4/adult molt (around 46 hours post L1 arrest) for each condition being assayed. Experiments contained multiple conditions (including the control) that were tested at the same time for a total of 100 to 125 plates–each containing a single hermaphrodite. These plates were banded together in stacks of five, placed in shoeboxes, and shifted at 48 hours post L1 arrest to 29°C for 24 hours. When the experiment called for male and hermaphrodite worms to be stressed together, age-matched pairs of males and hermaphrodites were used. After 24 hours of heat stress, the worms were returned to 20°C and allowed to recover. The numbers of eggs, both fertilized and unfertilized, and larvae produced by each worm were recorded daily. Worms were deemed to have recovered fecundity if they produced live progeny in the first 120 hours of recovery. See S1 Table for raw experimental data including numbers of independent trials and worms tested in each trial.

### Conditioning plates

*C*. *remanei* and *C*. *brenneri* produce large numbers of males. In other strains, males were generated by subjecting mid-L4 hermaphrodites to heat stress at 31°C for 3 hours and subsequently maintained by mating. To scent plates, males were segregated from hermaphrodites as L4 larvae and singled as one-day-old virgin adults. Unless otherwise noted, males were left on plates for 24 hours to deposit a scent and subsequently removed. Wild type and acox-1 hermaphrodites aged 24 hours post L1 arrest were used to condition plates until 52 hours post L1 arrest. This time span corresponds to the developmental stages from mid L3 larvae to young adult [47].

### Experiments with purified ascarosides

Purified ascr#3 and ascr#10 were a generous gift from Frank C. Schroeder (Cornell University). For recovery of fecundity experiments, ascarosides were diluted in 10% ethanol and applied to Noble agar (US Biological) NGM plates in a total volume of 100 uL. The concentrations listed represent the total amount of ascaroside applied to each plate. A 10% solution of ethanol alone was used as the control. The diluted ascarosides were spread on the plate with a glass rod and allowed to absorb into the agar overnight at 20°C. The next day, these plates were seeded with a 1:100 dilution of an overnight culture of OP50 bacteria and incubated at 20°C. The plates were used the following morning in heat stress and recovery experiments as described above.

### Experiments with *daf-7*

CB1372 and DA2202 strains were treated in the same manner. A one-hour egg lay was used to produce synchronous populations that were maintained at 16°C for 54 hours–until the worms were in the mid L3 larval stage. They were next transferred to 20°C for 16 hours until the young adult stage equivalent to that of N2 worms (raised at 20°C) at 48 hours post L1 arrest. This was confirmed by counting oocytes in the gonad [8]. Supplemental experiments used only DA2202 raised until young adulthood at 20°C (S12 Fig).

### Sperm guidance experiments with mCherry-labeled sperm

For experiments using total male scent, a synchronized population of EG4883 hermaphrodites [29] was raised on small population plates at 20°C until they were 48 hours post L1 arrest. Worms were transferred to either control plates or plates conditioned with four young males. For heat stress experiments, small population plates were shifted to 29°C for 24 hours.

For sperm guidance experiments with single ascarosides and cocktails of two ascarosides, the ascarosides were diluted in water and hermaphrodites were singled onto prepared plates in the same manner as heat stress and recovery experiments. These were single-blind experiments. In all sperm guidance experiments, worms were immobilized with sodium azide and mounted on 2% agarose pads. Images were taken with a Retiga 2000R camera mounted on a Leica DM5000B compound microscope and analyzed using ImageJ software. Individual images were stitched together using the MosaicJ plug-in [48]. Analyze Particles was used to count fluorescent sperm. An automatic thresholding program (MaxEntropy) [49] was used to determine image thresholds.

### Mating experiments with *C*. *remanei*

L4 *C*. *remanei* males were isolated from a mixed population and allowed to develop at 20°C overnight, after which they were used to condition half of the prepared mating plates (NGM plates with 5uL of a 1:100 dilution of an overnight culture of OP50 kept at 20°C overnight) for 24 hours. From a synchronous population (~40 hours post L1 arrest), females were moved to separate plates in groups of 20–30. At 48 hours post L1 arrest, they were singled onto either control or male-conditioned plates. Matings started 16 hours later, to ensure that both males and females were receptive. A single male was placed on top of an age-matched female in the shape of an X. Matings were short (10 minutes) to make multiple matings unlikely [23] and to ensure that males did not have a chance to deposit much scent on plates (S5 Fig). After 10 minutes, males were removed and the females were examined for the presence of a copulatory plug [50, 51]. All females, whether or not a copulatory plug was detected, were kept at 20°C and transferred to fresh, seeded NGM plates daily; fertilized eggs and larvae were counted.

## Supporting Information

S1 Fig Fraction of recovered hermaphrodites that have males in their broods. At 20°C the average male frequency in *C*. *elegans* is ≤ 0.002 [19]. The control is higher because heat stress increases the frequency of males [44]. Mating with unstressed males (column 2) produced male offspring in every brood. When males and hermaphrodites were stressed together (column 3), 40% of recovered hermaphrodites did not produce males in their broods suggesting that no matings took place. When males and hermaphrodites were stressed separately and allowed to recover together, almost 80% of offspring production occurred without mating. Consistent with this analysis of mating during recovery, brood sizes are higher for conditions where mating is possible. Average brood sizes are: control = 2.3, plus unstressed male = 46.5, hermaphrodite and male stressed together = 11.1, hermaphrodite and male stressed separately but recovered together = 4.4, and male or hermaphrodite or L3 scent = 2.7 (see also S7 Fig). Numbers above the bars represent total number of hermaphrodites that recovered fecundity, that is, the total number of broods examined in each column. Broods considered in this figure were derived from experiments in Fig 1 and S2 Fig.
(PDF)

S2 Fig Other tests of the effects of scent on reproductive recovery. We did not see a statistically significant difference in recovery when hermaphrodites and males were stressed separately and recovered together on plates scented with either hermaphrodite or male scent (column 2 compared to column 3 *P* = 0.77, binomial test). Scent from L3 larval worms did not improve recovery above background (column 4 compared to control *P* = 0.51, binomial test). Error bars denote ±SD among separate trials. Results described by white columns are from data presented in Fig 1B. See S1 Table for numbers of independent trials and worms tested in each trial.
(PDF)

S3 Fig Effects on recovery of fecundity of exposure to male scent during or after heat stress. Hermaphrodites were placed on male-scented plates during the 24 hours of heat stress and transferred to unscented plates for recovery or subjected to heat stress on unscented plates and transferred to male-scented plates for recovery. Neither regimen was as effective as maintaining the worms on male-scented plates during both stress and recovery. Results described by white columns are from data presented in Fig 1B. See S1 Table for numbers of independent trials and worms tested in each trial.
(PDF)

S4 Fig A cocktail composed of 5 fmol ascr#3 and 5 fmol ascr#10 is indistinguishable from the control. When the ratio of ascr#3 and ascr#10 was equal, recovery was no better than the control (*P* = 0.48, binomial test). Results described by white columns are from data presented in Fig 2. See S1 Table for numbers of independent trials and worms tested in each trial.
(PDF)

S5 Fig Self-recovery of fecundity after heat stress is improved when males have scented the plates for more than 12 hours. We allowed males to scent the plates used for heat stress and recovery of hermaphrodites for increasing amounts of time: less than 12 hours (10 minutes– 24 plates, 2 hours– 25 plates, and 12 hours– 25 plates, for a total of 74 plates), 16 hours (25 plates) and 48 hours (50 plates). Error bars denote ±SD among separate trials. Results described by white columns are from data presented in Fig 1B. See S1 Table for numbers of independent trials and worms tested in each trial.
(PDF)

S6 Fig Effects of different concentrations of ascr#3 and ascr#10. Error bars denote ±SD among separate trials. Hermaphrodites recovered fecundity significantly better with 10 fmol of ascr#3 than 2 fmol of ascr#3 (*P* = 5.1 x 10^−4^, binomial test). Results described by white columns are from data presented in Fig 2. The dashed line represents the recovery of fecundity of hermaphrodites on plates with male scent (data from Fig 1B). See S1 Table for numbers of trials and worms tested in each trial.
(PDF)

S7 Fig Brood sizes for the conditions in Fig 1B. Control and male scent data are the same data presented in Fig 4B. Only the brood sizes of males and hermaphrodites stressed together are significantly different from control (*P* = 1.4 X 10^−6^, Kolmogorov-Smirnov test Bonferroni corrected for five comparisons) because under that condition mating can take place (S1 Fig). Red bars indicate median values. Brood size means are: control = 2.3, males and hermaphrodites stressed together = 11.1, male-scented = 2.6, hermaphrodite-scented = 2.9, *daf-22* male-scented = 2.5, and *acox-1* hermaphrodite-scented = 2.2. See S2 Table for numbers of independent trials and worms tested in each trial.
(PDF)

S8 Fig Male scent does not change the dynamics of recovery of fecundity after heat stress. Hermaphrodites were monitored for 120 hours at 20°C after heat stress that lasted 24 hours at 29°C. The day that recovery progeny were first detected was noted for each recovering worm. Fractions of recovered hermaphrodites were normalized to the total of recovered hermaphrodites for each condition. See S2 Table for numbers of independent trials and worms tested in each trial.
(PDF)

S9 Fig Census of oocytes in the proximal gonad and embryos in the uterus for worms shifted to 29°C at 48 hours post L1 arrest on unscented or male-scented plates. See S2 Table for numbers of independent trials and worms tested in each trial.
(PDF)

S10 Fig EG4883, a strain with mCherry-marked sperm, recovers fecundity as well as N2. Recovery of fecundity of EG4883 is not significantly different from N2: EG4883 compared to N2 on control plates (white bar, data from Fig 1B) *P* = 0.4, binomial test; EG4883 compared to N2 on ascaroside control plates (white bar, data from Fig 2) *P* = 0.9, binomial test; and EG4883 compared to N2 on plates with male ascaroside cocktail (white bar, data from Fig 2) *P* = 0.13, binomial test. EG4883 on male cocktail plates recovered fecundity significantly better than on ascaroside control plates *P* = 9.8 x 10^−7^, binomial test. These experiments used *singled* hermaphrodites on plates with male cocktail diluted in water. See S1 Table for numbers of independent trials and worms tested in each trial.
(PDF)

S11 Fig Representative image of the gonad (with mCherry-labeled spermatids) in a hermaphrodite worm shifted to 31°C. When hermaphrodites were shifted to 31°C at 48 hours post L1 arrest, ovulation did not occur and all spermatids remained in the proximal gonad. The vulva is marked with a triangle. Anterior is to the left and ventral is down.
(PDF)

S12 Fig Larval growth conditions affect recovery from heat stress. When N2 worms were grown at 16°C until the end of L2 stage and shifted to 20°C until early adulthood in the same way that *daf-7* hermaphrodites were treated in Fig 7, they recovered fecundity better after heat stress (A). The N2 hermaphrodites treated in this way still responded to male scent (*P* = 0.05, binomial test). Results described by white columns are from data presented in Fig 1B. Conversely, when *daf-7*(*e1372*);*gpa-4p*::*daf-7* worms were grown at 20°C, they recovered fecundity after heat stress less well than the same worms raised at 16°C until the end of L2 stage and shifted to 20°C until young adulthood (B). In this strain the *daf-7* gene product is not inducible and the worms do not respond to male scent (comparing control and male scent for worms raised at 20°C, *P* = 1.0, binomial test; comparing control and male scent for worms raised at 16°C then 20°C, *P* = 0.56, binomial test). Results described by white columns are from data presented in Fig 7. Error bars denote ±SD among separate trials. See S1 Table for numbers of independent trials and worms tested in each trial.
(PDF)

S1 Table Summary of experiments and numbers of animals tested in this study (recovery of fecundity data). (XLSX)

S2 Table Summary of experiments and numbers of animals tested in this study (brood size, sperm guidance, and related data). (XLSX)
