# Supplementary material for: Sex Pheromones of C. elegans Males Prime the Female Reproductive System and Ameliorate the Effects of Heat Stress
Source: PLoS Genet. 2015 Dec 8;11(12):e1005729. doi: 10.1371/journal.pgen.1005729 (PMC4672928; doi:10.1371/journal.pgen.1005729)
Supplement: S1 Fig — At 20°C the average male frequency in C. elegans is ≤ 0.002 [19]. The control is higher because heat stress increases the frequency of males [44]. Mating with unstressed males (column 2) produced male offspring in every brood. When males and hermaphrodites were stressed together (column 3), 40% of recovered hermaphrodites did not produce males in their broods suggesting that no matings took place. When males and hermaphrodites were stressed separately and allowed to recover together, almost 80% of offspring production occurred without mating. Consistent with this analysis of mating during recovery, brood sizes are higher for conditions where mating is possible. Average brood sizes are: control = 2.3, plus unstressed male = 46.5, hermaphrodite and male stressed together = 11.1, hermaphrodite and male stressed separately but recovered together = 4.4, and male or hermaphrodite or L3 scent = 2.7 (see also S7 Fig). Numbers above the bars represent total number of hermaphrodites that recovered fecundity, that is, the total number of broods examined in each column. Broods considered in this figure were derived from experiments in Fig 1 and S2 Fig. (PDF) [file pgen.1005729.s001.pdf]

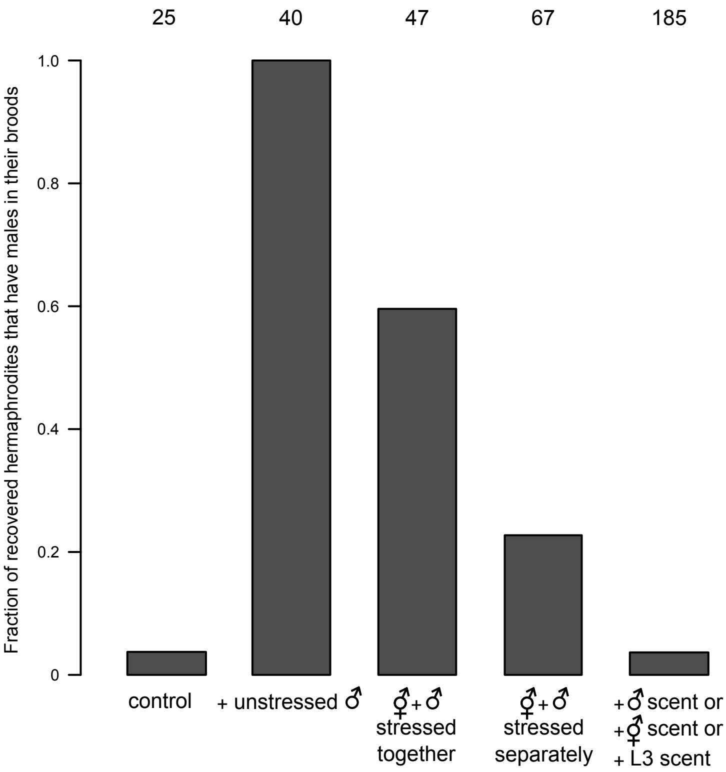

S1 Fig. Fraction of recovered hermaphrodites that have males in their broods. At 20°C the average male frequency in *C. elegans* is ≤ 0.002 [19]. The control is higher because heat stress increases the frequency of males [44]. Mating with unstressed males (column 2) produced male offspring in every brood. When males and hermaphrodites were stressed together (column 3), 40% of recovered hermaphrodites did not produce males in their broods suggesting that no matings took place. When males and hermaphrodites were stressed separately and allowed to recover together, almost 80% of offspring production occurred without mating. Consistent with this analysis of mating during recovery, brood sizes are higher for conditions where mating is possible. Average brood sizes are: control=2.3, plus unstressed male=46.5, hermaphrodite and male stressed together=11.1, hermaphrodite and male stressed separately but recovered together=4.4, and male or hermaphrodite or L3 scent=2.7 (see also S7 Fig). Numbers above the bars represent total number of hermaphrodites that recovered fecundity, that is the total number of broods examined in each column. Broods considered in this figure were derived from experiments in Fig. 1 and S2 Fig.
