# Supplementary material for: Sex Pheromones of C. elegans Males Prime the Female Reproductive System and Ameliorate the Effects of Heat Stress
Source: PLoS Genet. 2015 Dec 8;11(12):e1005729. doi: 10.1371/journal.pgen.1005729 (PMC4672928; doi:10.1371/journal.pgen.1005729)
Supplement: S2 Fig — We did not see a statistically significant difference in recovery when hermaphrodites and males were stressed separately and recovered together on plates scented with either hermaphrodite or male scent (column 2 compared to column 3 P = 0.77, binomial test). Scent from L3 larval worms did not improve recovery above background (column 4 compared to control P = 0.51, binomial test). Error bars denote ±SD among separate trials. Results described by white columns are from data presented in Fig 1B. See S1 Table for numbers of independent trials and worms tested in each trial. (PDF) [file pgen.1005729.s002.pdf]

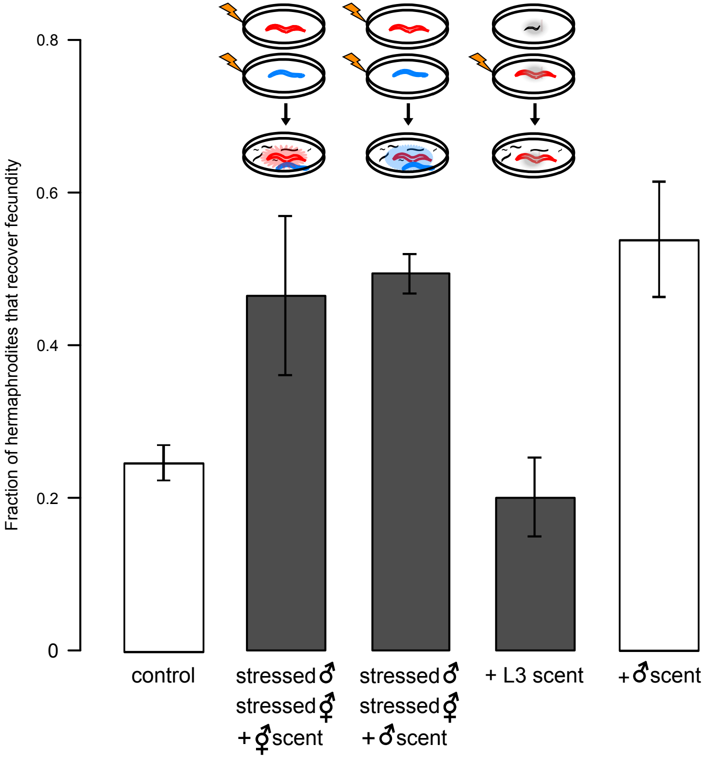

S2 Fig. Other tests of the effects of scent on reproductive recovery. We did not see a statistically significant difference in recovery when hermaphrodites and males were stressed separately and recovered together on plates scented with either hermaphrodite or male scent (column 2 compared to column 3 $P = 0.77$, binomial test). Scent from L3 larval worms did not improve recovery above background (column 4 compared to control $P = 0.51$, binomial test). Error bars denote ±SD among separate trials. Results described by white columns are from data presented in Fig. 1B. See S1 Table for numbers of independent trials and worms tested in each trial.
