# Supplementary material for: Sex Pheromones of C. elegans Males Prime the Female Reproductive System and Ameliorate the Effects of Heat Stress
Source: PLoS Genet. 2015 Dec 8;11(12):e1005729. doi: 10.1371/journal.pgen.1005729 (PMC4672928; doi:10.1371/journal.pgen.1005729)
Supplement: S3 Fig — Hermaphrodites were placed on male-scented plates during the 24 hours of heat stress and transferred to unscented plates for recovery or subjected to heat stress on unscented plates and transferred to male-scented plates for recovery. Neither regimen was as effective as maintaining the worms on male-scented plates during both stress and recovery. Results described by white columns are from data presented in Fig 1B. See S1 Table for numbers of independent trials and worms tested in each trial. (PDF) [file pgen.1005729.s003.pdf]

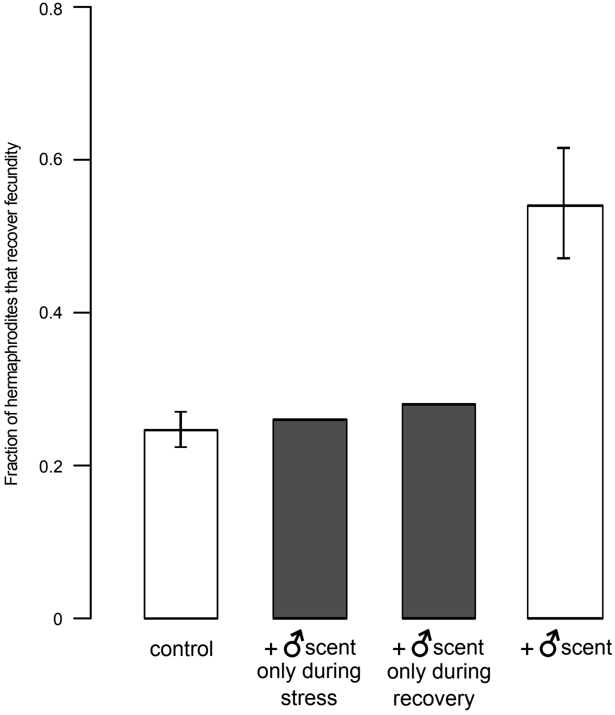

S3 Fig. Effects on recovery of fecundity of exposure to male scent during or after heat stress. Hermaphrodites were placed on male-scented plates during the 24 hours of heat stress and transferred to unscented plates for recovery or subjected to heat stress on unscented plates and transferred to male-scented plates for recovery. Neither regimen was as effective as maintaining the worms on male-scented plates during both stress and recovery. Results described by white columns are from data presented in Fig. 1B. See S1 Table for numbers of independent trials and worms tested in each trial.
