# Supplementary material for: Sex Pheromones of C. elegans Males Prime the Female Reproductive System and Ameliorate the Effects of Heat Stress
Source: PLoS Genet. 2015 Dec 8;11(12):e1005729. doi: 10.1371/journal.pgen.1005729 (PMC4672928; doi:10.1371/journal.pgen.1005729)
Supplement: S4 Fig — When the ratio of ascr#3 and ascr#10 was equal, recovery was no better than the control (P = 0.48, binomial test). Results described by white columns are from data presented in Fig 2. See S1 Table for numbers of independent trials and worms tested in each trial. (PDF) [file pgen.1005729.s004.pdf]

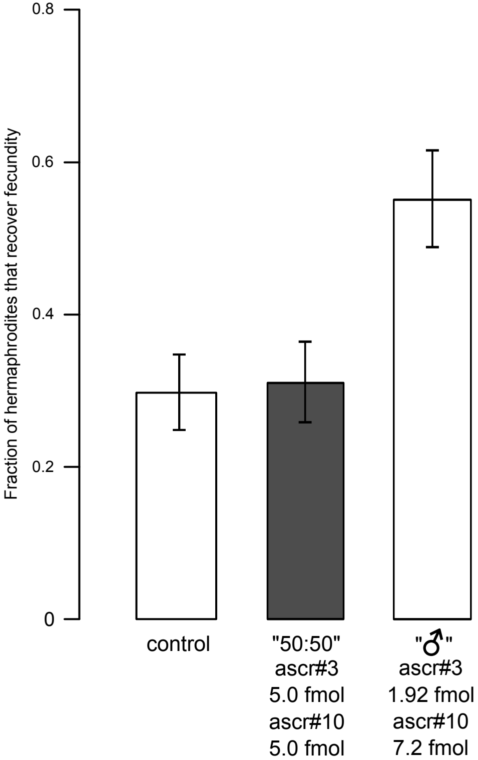

S4 Fig. A cocktail composed of 5 fmol ascr#3 and 5 fmol ascr#10 is indistinguishable from the control. When the ratio of ascr#3 and ascr#10 was equal, recovery was no better than the control ($P = 0.48$, binomial test). Results described by white columns are from data presented in Fig. 2. See S1 Table for numbers of independent trials and worms tested in each trial.
