# Supplementary material for: Sex Pheromones of C. elegans Males Prime the Female Reproductive System and Ameliorate the Effects of Heat Stress
Source: PLoS Genet. 2015 Dec 8;11(12):e1005729. doi: 10.1371/journal.pgen.1005729 (PMC4672928; doi:10.1371/journal.pgen.1005729)
Supplement: S5 Fig — We allowed males to scent the plates used for heat stress and recovery of hermaphrodites for increasing amounts of time: less than 12 hours (10 minutes– 24 plates, 2 hours– 25 plates, and 12 hours– 25 plates, for a total of 74 plates), 16 hours (25 plates) and 48 hours (50 plates). Error bars denote ±SD among separate trials. Results described by white columns are from data presented in Fig 1B. See S1 Table for numbers of independent trials and worms tested in each trial. (PDF) [file pgen.1005729.s005.pdf]

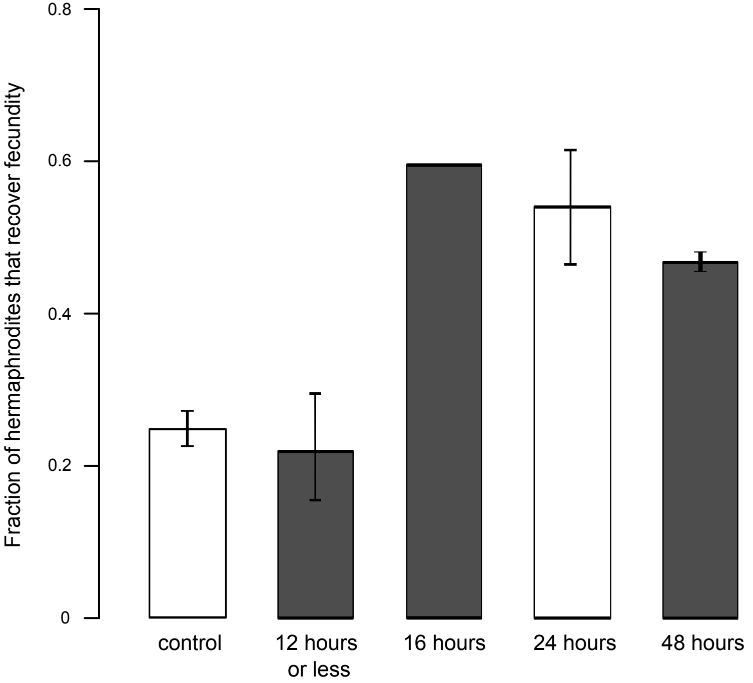

S5 Fig. Self-recovery of fecundity after heat stress is improved when males have scented the plates for more than 12 hours. We allowed males to scent the plates used for heat stress and recovery of hermaphrodites for increasing amounts of time: less than 12 hours (10 minutes - 24 plates, 2 hours - 25 plates, and 12 hours - 25 plates, for a total of 74 plates), 16 hours (25 plates) and 48 hours (50 plates). Error bars denote ±SD among separate trials. Results described by white columns are from data presented in Fig. 1B. See S1 Table for numbers of independent trials and worms tested in each trial.
