# Supplementary material for: Sex Pheromones of C. elegans Males Prime the Female Reproductive System and Ameliorate the Effects of Heat Stress
Source: PLoS Genet. 2015 Dec 8;11(12):e1005729. doi: 10.1371/journal.pgen.1005729 (PMC4672928; doi:10.1371/journal.pgen.1005729)
Supplement: S6 Fig — Error bars denote ±SD among separate trials. Hermaphrodites recovered fecundity significantly better with 10 fmol of ascr#3 than 2 fmol of ascr#3 (P = 5.1 x 10^−4^, binomial test). Results described by white columns are from data presented in Fig 2. The dashed line represents the recovery of fecundity of hermaphrodites on plates with male scent (data from Fig 1B). See S1 Table for numbers of trials and worms tested in each trial. (PDF) [file pgen.1005729.s006.pdf]

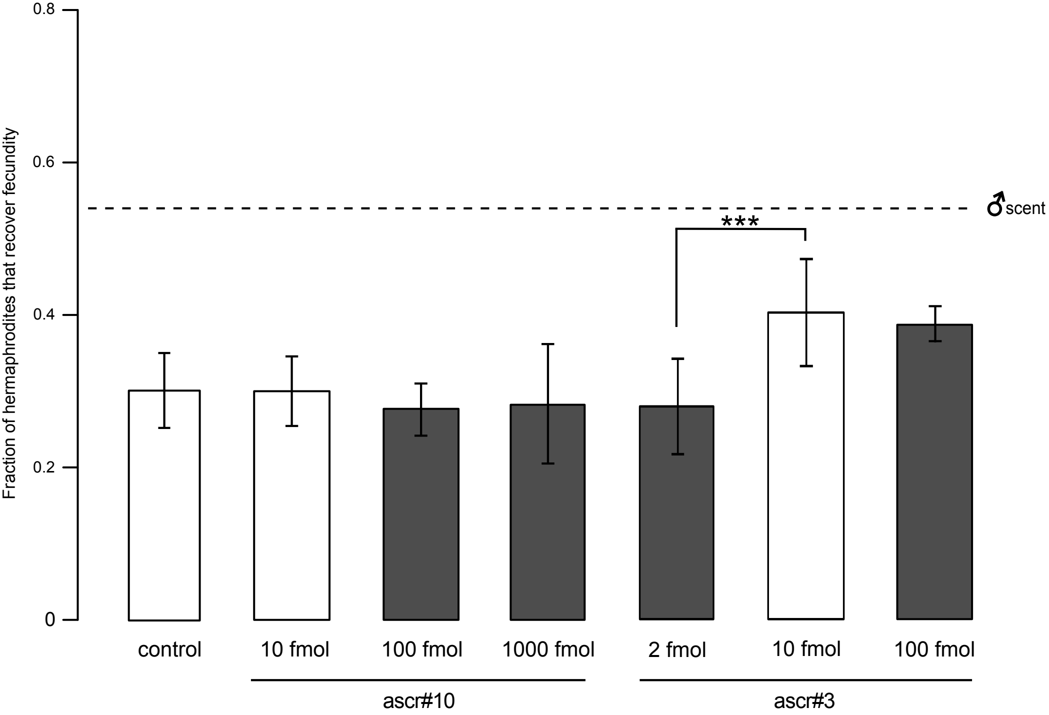

S6 Fig. Effects of different concentrations of ascr#3 and ascr#10. Error bars denote ±SD among separate trials. Hermaphrodites recovered fecundity significantly better with 10 fmol of ascr#3 than 2 fmol of ascr#3 ($P = 5.1 \times 10^{-4}$, binomial test). Results described by white columns are from data presented in Fig. 2. The dashed line represents the recovery of fecundity of hermaphrodites on plates with male scent (data from Fig. 1B). See S1 Table for numbers of trials and worms tested in each trial.
