# Supplementary material for: Sex Pheromones of C. elegans Males Prime the Female Reproductive System and Ameliorate the Effects of Heat Stress
Source: PLoS Genet. 2015 Dec 8;11(12):e1005729. doi: 10.1371/journal.pgen.1005729 (PMC4672928; doi:10.1371/journal.pgen.1005729)
Supplement: S7 Fig — Control and male scent data are the same data presented in Fig 4B. Only the brood sizes of males and hermaphrodites stressed together are significantly different from control (P = 1.4 X 10−6, Kolmogorov-Smirnov test Bonferroni corrected for five comparisons) because under that condition mating can take place (S1 Fig). Red bars indicate median values. Brood size means are: control = 2.3, males and hermaphrodites stressed together = 11.1, male-scented = 2.6, hermaphrodite-scented = 2.9, daf-22 male-scented = 2.5, and acox-1 hermaphrodite-scented = 2.2. See S2 Table for numbers of independent trials and worms tested in each trial. (PDF) [file pgen.1005729.s007.pdf]

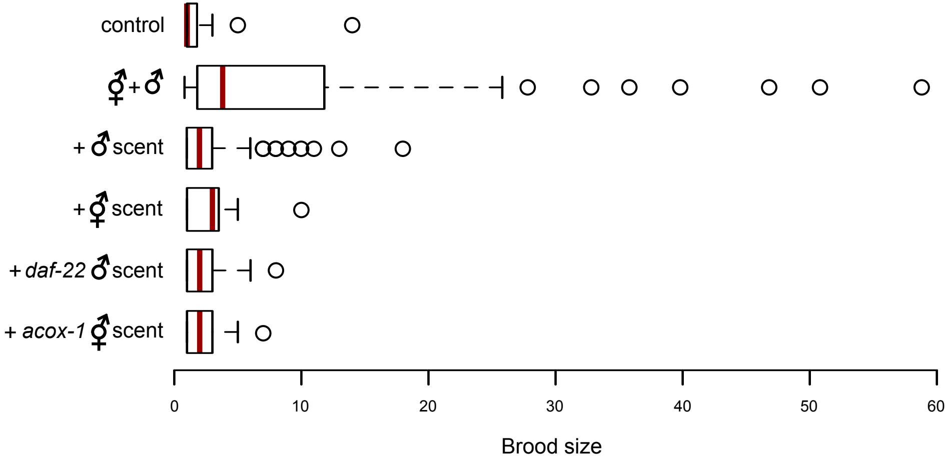

S7 Fig. Brood sizes for the conditions in Fig 1B. Control and male scent data are the same data presented in Fig. 4B. Only the brood sizes of males and hermaphrodites stressed together are significantly different from control ($P = 1.4 \times 10^{-6}$, Kolmogorov-Smirnov test Bonferroni corrected for five comparisons) because under that condition mating can take place (S1 Fig). Red bars indicate median values. Brood size means are: control=2.3, males and hermaphrodites stressed together=11.1, male-scented=2.6, hermaphrodite-scented=2.9, *daf-22* male-scented=2.5, and *acox-1* hermaphrodite-scented=2.2. See S2 Table for numbers of independent trials and worms tested in each trial.
