# Supplementary material for: Sex Pheromones of C. elegans Males Prime the Female Reproductive System and Ameliorate the Effects of Heat Stress
Source: PLoS Genet. 2015 Dec 8;11(12):e1005729. doi: 10.1371/journal.pgen.1005729 (PMC4672928; doi:10.1371/journal.pgen.1005729)
Supplement: S8 Fig — Hermaphrodites were monitored for 120 hours at 20°C after heat stress that lasted 24 hours at 29°C. The day that recovery progeny were first detected was noted for each recovering worm. Fractions of recovered hermaphrodites were normalized to the total of recovered hermaphrodites for each condition. See S2 Table for numbers of independent trials and worms tested in each trial. (PDF) [file pgen.1005729.s008.pdf]

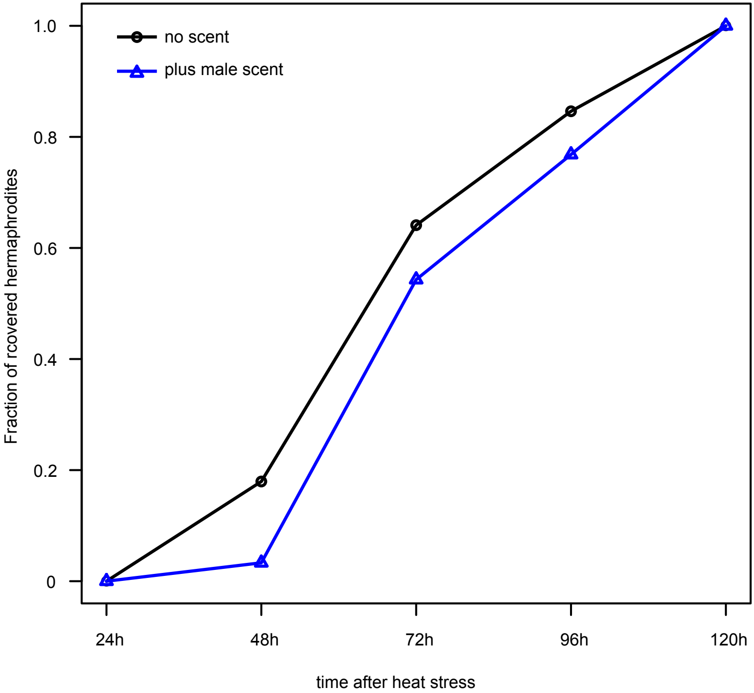

S8 Fig. Male scent does not change the dynamics of recovery of fecundity after heat stress. Hermaphrodites were monitored for 120 hours at 20°C after heat stress that lasted 24 hours at 29°C. The day that recovery progeny were first detected was noted for each recovering worm. Fractions of recovered hermaphrodites were normalized to the total of recovered hermaphrodites for each condition. See S2 Table for numbers of independent trials and worms tested in each trial.
