# Supplementary material for: Sex Pheromones of C. elegans Males Prime the Female Reproductive System and Ameliorate the Effects of Heat Stress
Source: PLoS Genet. 2015 Dec 8;11(12):e1005729. doi: 10.1371/journal.pgen.1005729 (PMC4672928; doi:10.1371/journal.pgen.1005729)
Supplement: S9 Fig — See S2 Table for numbers of independent trials and worms tested in each trial. (PDF) [file pgen.1005729.s009.pdf]

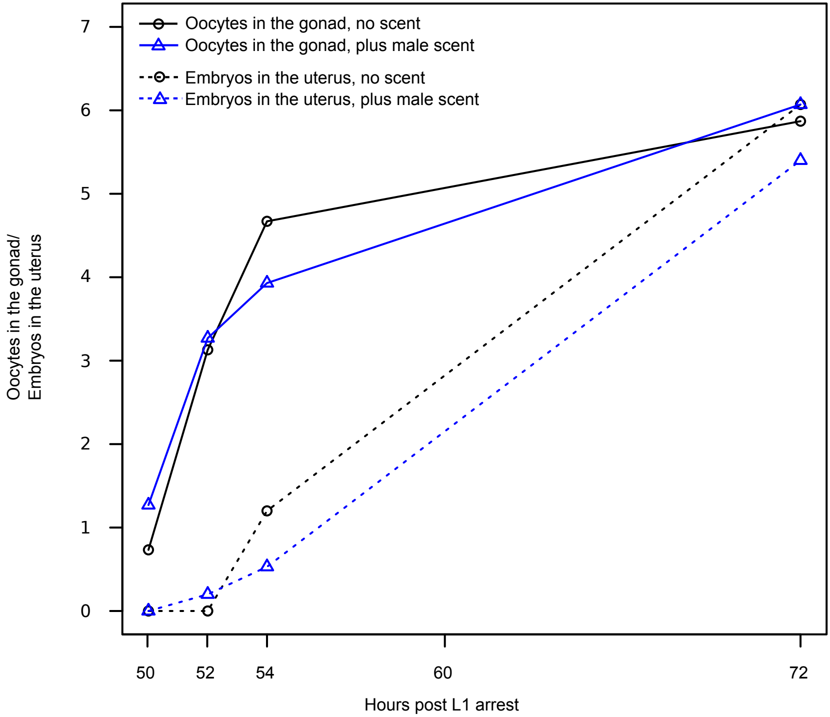

S9 Fig. Census of oocytes in the proximal gonad and embryos in the uterus for worms shifted to 29°C at 48 hours post L1 arrest on unscented or male-scented plates. See S2 Table for numbers of independent trials and worms tested in each trial.
