# Supplementary material for: Sex Pheromones of C. elegans Males Prime the Female Reproductive System and Ameliorate the Effects of Heat Stress
Source: PLoS Genet. 2015 Dec 8;11(12):e1005729. doi: 10.1371/journal.pgen.1005729 (PMC4672928; doi:10.1371/journal.pgen.1005729)
Supplement: S10 Fig — Recovery of fecundity of EG4883 is not significantly different from N2: EG4883 compared to N2 on control plates (white bar, data from Fig 1B) P = 0.4, binomial test; EG4883 compared to N2 on ascaroside control plates (white bar, data from Fig 2) P = 0.9, binomial test; and EG4883 compared to N2 on plates with male ascaroside cocktail (white bar, data from Fig 2) P = 0.13, binomial test. EG4883 on male cocktail plates recovered fecundity significantly better than on ascaroside control plates P = 9.8 x 10^−7^, binomial test. These experiments used singled hermaphrodites on plates with male cocktail diluted in water. See S1 Table for numbers of independent trials and worms tested in each trial. (PDF) [file pgen.1005729.s010.pdf]

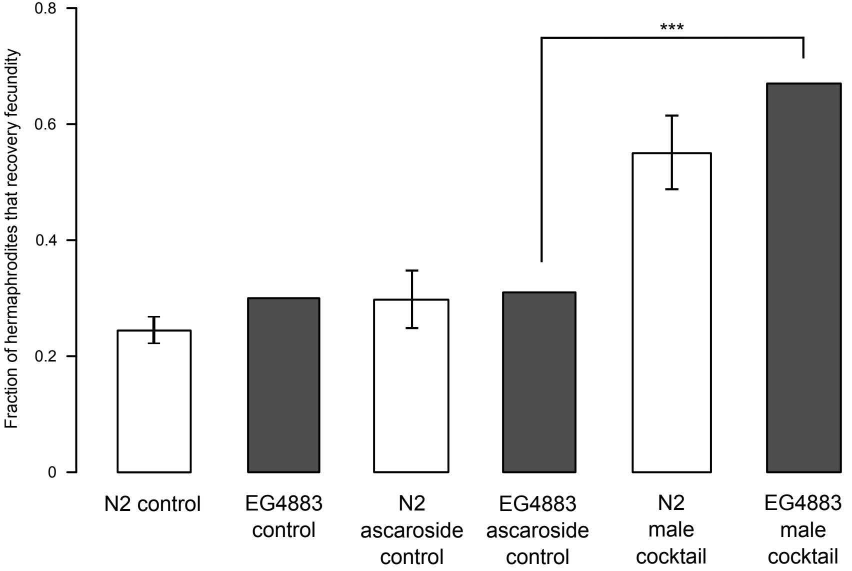

S10 Fig. EG4883, a strain with mCherry-marked sperm, recovers fecundity as well as N2. Recovery of fecundity of EG4883 is not significantly different from N2: EG4883 compared to N2 on control plates (white bar, data from Fig. 1B) $P = 0.4$, binomial test; EG4883 compared to N2 on ascaroside control plates (white bar, data from Fig. 2) $P = 0.9$, binomial test; and EG4883 compared to N2 on plates with male ascaroside cocktail (white bar, data from Fig. 2) $P = 0.13$, binomial test. EG4883 on male cocktail plates recovered fecundity significantly better than on ascaroside control plates $P = 9.8 \times 10^{-7}$, binomial test. These experiments used *singled* hermaphrodites on plates with male cocktail diluted in water. See S1 Table for numbers of independent trials and worms tested in each trial.
