# Supplementary material for: Sex Pheromones of C. elegans Males Prime the Female Reproductive System and Ameliorate the Effects of Heat Stress
Source: PLoS Genet. 2015 Dec 8;11(12):e1005729. doi: 10.1371/journal.pgen.1005729 (PMC4672928; doi:10.1371/journal.pgen.1005729)
Supplement: S11 Fig — When hermaphrodites were shifted to 31°C at 48 hours post L1 arrest, ovulation did not occur and all spermatids remained in the proximal gonad. The vulva is marked with a triangle. Anterior is to the left and ventral is down. (PDF) [file pgen.1005729.s011.pdf]

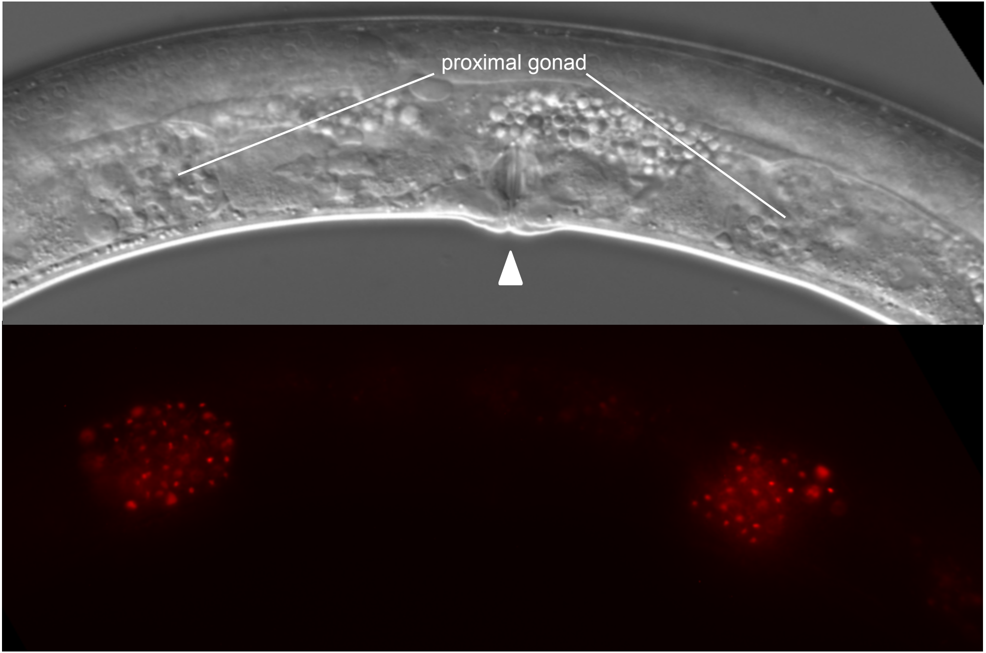

S11 Fig. Representative image of the gonad (with mCherry-labeled spermatids) in a hermaphrodite worm shifted to 31°C. When hermaphrodites were shifted to 31°C at 48 hours post L1 arrest, ovulation did not occur and all spermatids remained in the proximal gonad. The vulva is marked with a triangle. Anterior is to the left and ventral is down.
