# Supplementary material for: Sex Pheromones of C. elegans Males Prime the Female Reproductive System and Ameliorate the Effects of Heat Stress
Source: PLoS Genet. 2015 Dec 8;11(12):e1005729. doi: 10.1371/journal.pgen.1005729 (PMC4672928; doi:10.1371/journal.pgen.1005729)
Supplement: S12 Fig — When N2 worms were grown at 16°C until the end of L2 stage and shifted to 20°C until early adulthood in the same way that daf-7 hermaphrodites were treated in Fig 7, they recovered fecundity better after heat stress (A). The N2 hermaphrodites treated in this way still responded to male scent (P = 0.05, binomial test). Results described by white columns are from data presented in Fig 1B. Conversely, when daf-7(e1372);gpa-4p::daf-7 worms were grown at 20°C, they recovered fecundity after heat stress less well than the same worms raised at 16°C until the end of L2 stage and shifted to 20°C until young adulthood (B). In this strain the daf-7 gene product is not inducible and the worms do not respond to male scent (comparing control and male scent for worms raised at 20°C, P = 1.0, binomial test; comparing control and male scent for worms raised at 16°C then 20°C, P = 0.56, binomial test). Results described by white columns are from data presented in Fig 7. Error bars denote ±SD among separate trials. See S1 Table for numbers of independent trials and worms tested in each trial. (PDF) [file pgen.1005729.s012.pdf]

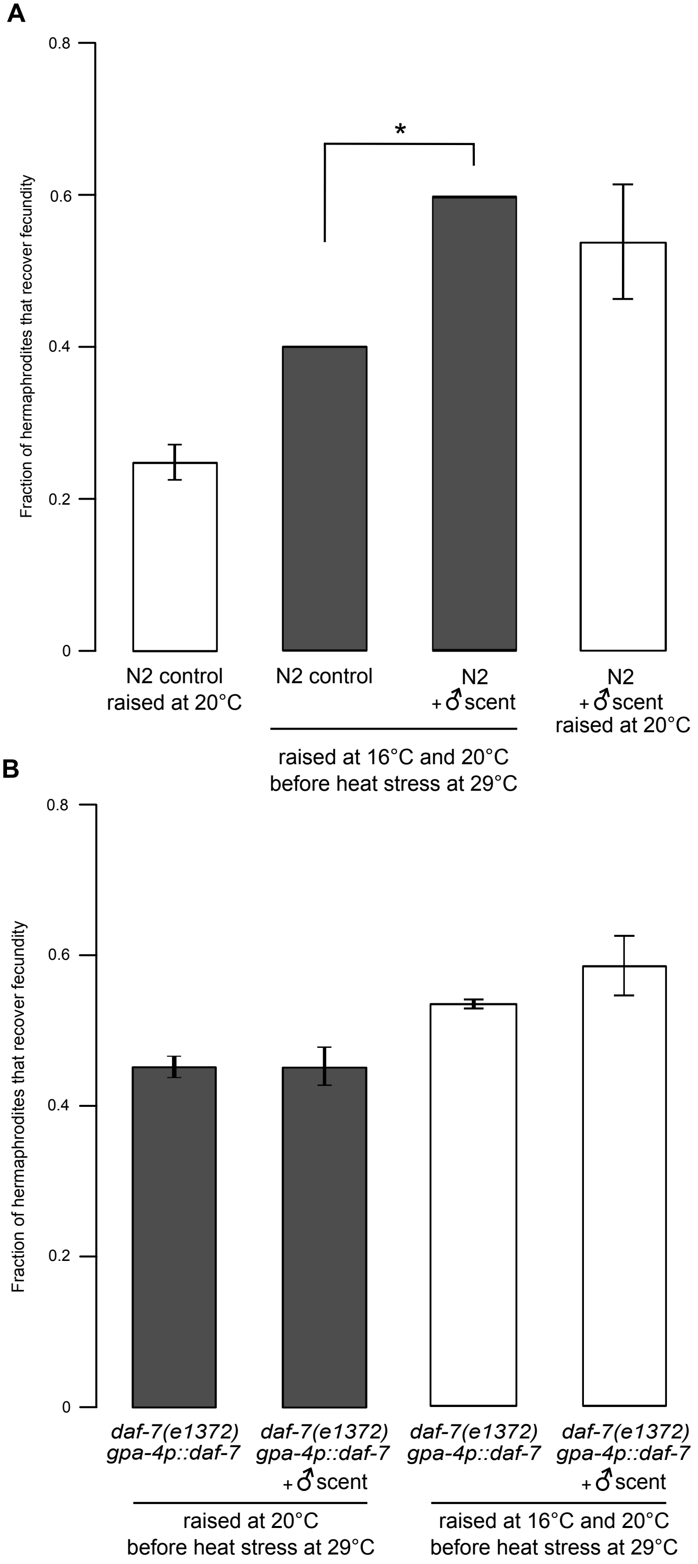

S12 Fig. Larval growth conditions affect recovery from heat stress. When N2 worms were grown at 16°C until the end of L2 stage and shifted to 20°C until early adulthood in the same way that *daf-7* hermaphrodites were treated in Fig. 7, they recovered fecundity better after heat stress (A). The N2 hermaphrodites treated in this way still responded to male scent ($P = 0.05$, binomial test). Results described by white columns are from data presented in Fig. 1B. Conversely, when *daf-7(e1372); gpa-4p::daf-7* worms were grown at 20°C, they recovered fecundity after heat stress less well than the same worms raised at 16°C until the end of L2 stage and shifted to 20°C until young adulthood (B). In this strain the *daf-7* gene product is not inducible and the worms do not respond to male scent (comparing control and male scent for worms raised at 20°C, $P = 1.0$, binomial test; comparing control and male scent for worms raised at 16°C then 20°C, $P = 0.56$, binomial test). Results described by white columns are from data presented in Fig. 7. Error bars denote ±SD among separate trials. See S1 Table for numbers of independent trials and worms tested in each trial.
